# Advances in KRAS-Targeted Therapies for Pancreatic Cancer

**DOI:** 10.3390/cancers18182961

**Published:** 2026-09-14

**Authors:** Supriya Peshin, Afra Zahid, Ehab Takrori, Zahra Hamedi, Shafia Rahman

**Affiliations:** 1 Division of Geriatrics, Department of Internal Medicine, University of Arkansas for Medical Sciences, Little Rock, AR 72204, USA; speshin@uams.edu; 2 Department of Internal Medicine, Allegheny Health Network, Pittsburgh, PA 15212, USA; afra.zahid@ahn.org; 3 Department of Internal Medicine, Alfaisal University, Riyadh 11533, Saudi Arabia; etakrori@gmail.com; 4 Division of Hematology and Oncology, Department of Medicine, University of California, Irvine, CA 92697, USA; zhamedi@hs.uci.edu; 5 Division of Medical Oncology, Department of Internal Medicine, The Ohio State University Comprehensive Cancer Center—James Cancer Hospital and Solove Research Institute, Colombus, OH 43210, USA

**Keywords:** pancreatic ductal adenocarcinoma (PDAC), KRAS-directed therapy, drug resistance, combination therapy, precision oncology

## Abstract

The incidence of pancreatic ductal adenocarcinoma (PDAC) is increasing globally, and PDAC remains one of the deadliest malignancies, primarily because of late-stage presentation at diagnosis and limited effective therapies. *KRAS* mutations are present in approximately 88–92% of PDAC cases, making *KRAS* an important therapeutic target in PDAC, despite its long-standing historical classification as being “undruggable.” This review comprehensively examines the role of *KRAS* in pancreatic cancer and summarizes both indirect and direct KRAS-targeted therapeutic strategies, including downstream pathway inhibition, metabolic targeting, and emerging direct KRAS inhibitors. The ongoing clinical trials are essential for establishing the efficacy of these emerging therapies, which are poised to gradually transition into clinical practice and ultimately improve patient outcomes. A major challenge with KRAS-targeted therapy is drug resistance, which occurs through both on-target and bypass mechanisms, but emerging combination strategies targeting parallel pathways show promise. Combination approaches involving chemotherapy, immunotherapy, PRMT5 inhibition, and tumor microenvironment targeting are being actively studied to overcome resistance. Overall, KRAS-directed therapies represent a significant and evolving advancement with the potential to improve outcomes in pancreatic cancer.

## Simple Summary

Pancreatic cancer remains one of the most difficult cancers to treat, and most tumors carry changes in a gene called *KRAS*. For many years, *KRAS* was considered impossible to target with drugs. Recent advances have led to the development of medicines that directly block specific *KRAS* mutations, as well as broader treatments that can inhibit several *KRAS* forms. This review explains how *KRAS* drives pancreatic cancer, summarizes the main direct and indirect treatment approaches, and discusses ongoing clinical trials. It also highlights a major challenge: tumors often develop resistance when KRAS inhibitors are used alone. Combining these drugs with chemotherapy, immunotherapy, or treatments aimed at other cancer pathways may produce stronger and longer-lasting responses. Although most approaches are still being tested, KRAS-directed therapy represents an important step toward more precise and effective treatment for patients with pancreatic cancer.

## 1. Introduction

Pancreatic Adenocarcinoma (PDAC) represents a growing global health burden, with its incidence increasing from 5.22 per 100,000 in 1990 to 6.57 per 100,000 in 2019 [1]. Currently the sixth leading cause of cancer-related death worldwide [2], pancreatic cancer is projected to become the second leading cause of cancer deaths in the United States by 2030 [3]. Thus, pancreatic cancer continues to represent a major challenge in oncology. PDAC carries a poor prognosis, with a 5-year overall survival rate of approximately 10–13% [2]. The dismal prognosis stems primarily from lack of early detection strategies and limited effective therapeutic options [4]. The asymptomatic nature of early-stage disease precludes timely detection, while the propensity for early systemic spread and limited therapeutic strategies further compound the poor outcome.

Pancreatic ductal adenocarcinoma (PDAC) accounts for 85% of all pancreatic cancers [4], with nearly all cases (95%) harboring mutationally activated *KRAS* oncogene [5]. The near-universal presence of activating *KRAS* mutation in PDAC position this oncogene as the most specific and defining genetic hallmark of the disease, establishing it as a critical therapeutic target for precision medicine approaches [6]. However, *KRAS* mutations were long considered to be ‘undruggable’ because they evaded pharmacological inhibition. This was attributed to the fact that the *KRAS* proteins lack prominent and accessible binding sites for small-molecule inhibitors other than the guanosine triphosphate (GTP)-binding pocket, which itself is difficult to target because of the high affinity of *KRAS* for GTP and the naturally high intracellular concentration of GTP in cells [7]. Due to this historical ‘undruggability’ of *KRAS*, there was greater focus on indirect strategies, rather than direct KRAS inhibition, and those indirect approaches yielded disappointing results in *KRAS*-mutant cancers.

There has been a recent breakthrough in KRAS-directed therapy through the work of Janes et al., who discovered a covalent compound, ARS-1620, that successfully inhibits the KRAS G12C [8]. This success has initiated an intense pursuit of inhibitors targeting the PDAC-prevalent KRAS mutants, particularly G12D, G12V, and G12R, with several promising candidates now entering clinical trials [9]. These developments hold major importance given the 5-year survival rate of PDAC patients of 13% [10].

The inability to directly target *KRAS* in the past has remained a major obstacle in the treatment of PDAC. This review aims to comprehensively describe the scope and challenges of KRAS-directed therapy for the treatment of pancreatic cancer and discuss how this treatment modality can influence the prognosis of PDAC patients in the future.

Clinical evidence and clinical trial information included in this review were verified through 29 July 2026. Trial identifiers, study phase, recruitment status, and study start dates were checked against ClinicalTrials.gov.(accessed on 10 September 2026) Peer-reviewed publications were prioritized for efficacy estimates when available, whereas data available only from conference abstracts or presentations are explicitly identified in the relevant tables.

## 2. Discussion

### 2.1. The Role of KRAS in Pancreatic Cancer

Pancreatic ductal adenocarcinoma is characterized by a well-defined genetic landscape dominated by four key driver mutations: activating mutation in *KRAS* oncogene (88–92%), and inactivating mutation in tumor suppressor genes *TP53* (61–74%), *CDKN2A* (16–44%), and *SMAD4* (20–22%) [2]. Among these, *KRAS* plays a central role because more than 90% of the cases of PDAC harbor *KRAS* mutations [5]. *KRAS* mutations are predominantly found at codon 12 (G12D, G12V, G12R) and are considered activating because they lead to constitutive activation of downstream pathways by locking the *KRAS* protein in the GTP-bound state [11]. This mutant *KRAS* is not only involved in tumor initiation, but also helps maintain tumor proliferation [12], which further adds to its importance as a potential target for the treatment of pancreatic cancer.

**Figure 1:**
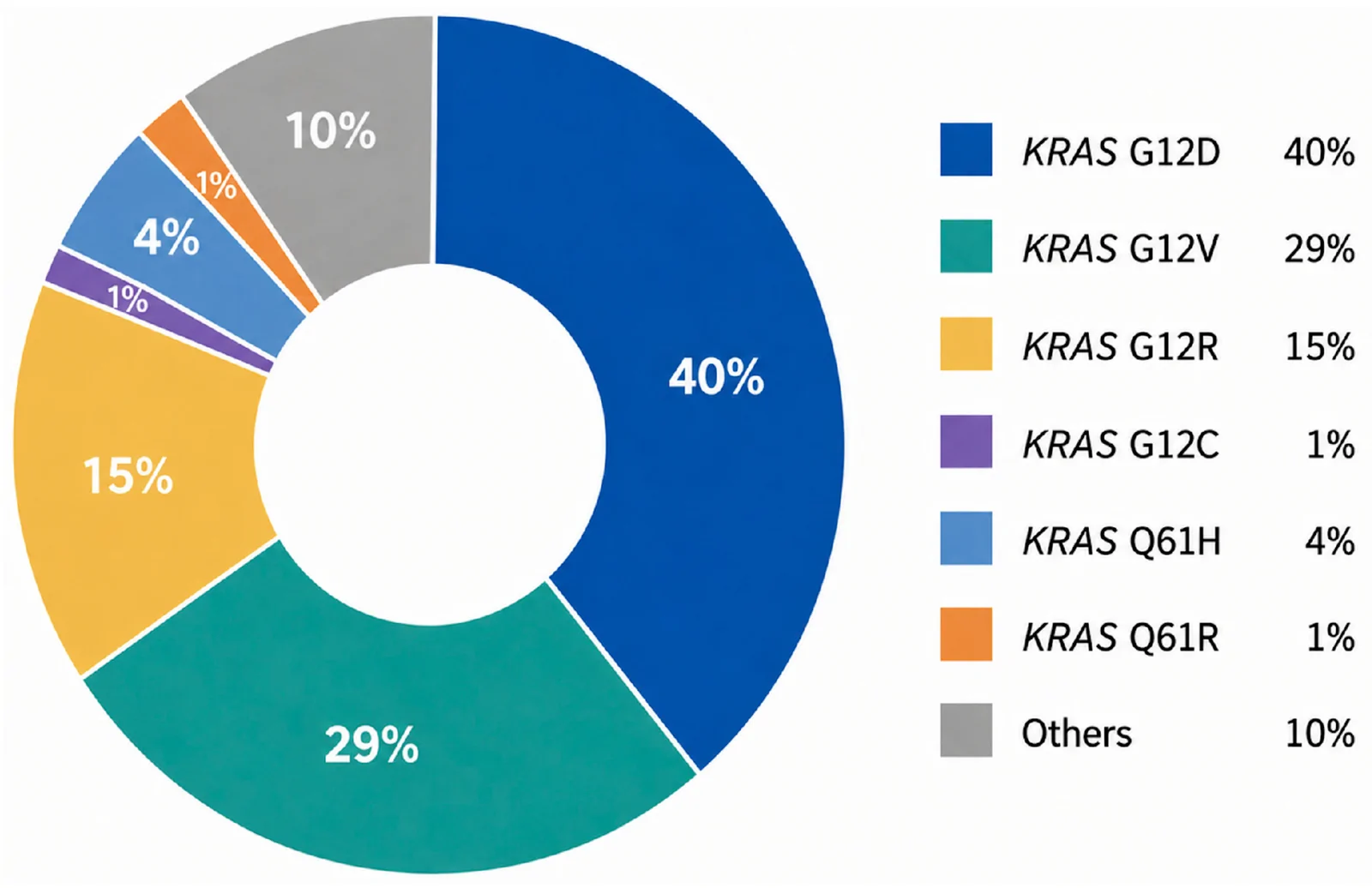
Distribution of *KRAS* Mutation Subtypes in PDAC. Approximate frequencies of the major *KRAS* variants reported in PDAC cohorts are shown, with G12D being the most common subtype, followed by G12V and G12R. Less frequent variants include G12C, Q61H, Q61R, and other uncommon *KRAS* alterations. Percentages represent approximate rounded frequencies and may vary slightly across studies because of differences in cohort characteristics and sequencing methods.

*KRAS* mutations not only drive tumor initiation and sustain cellular proliferation of the tumor cells but also play a key role in remodeling the tumor microenvironment by promoting accumulation of proliferating mesenchymal cells like fibroblasts and stellate cells [13]. Furthermore, the immune cells that infiltrate the pancreas, including immune-suppressing cells like regulatory T cells, myeloid-derived suppressor cells, and mast cells, also appear to be regulated by *KRAS* [13]. Hence, *KRAS* has a pivotal role in not only the development, but also the maintenance and progression of, pancreatic cancer. The frequencies of the major *KRAS* mutation subtypes in PDAC are summarized in Figure 1.

### 2.2. The KRAS Downstream Signaling Pathway

**Figure 2:**
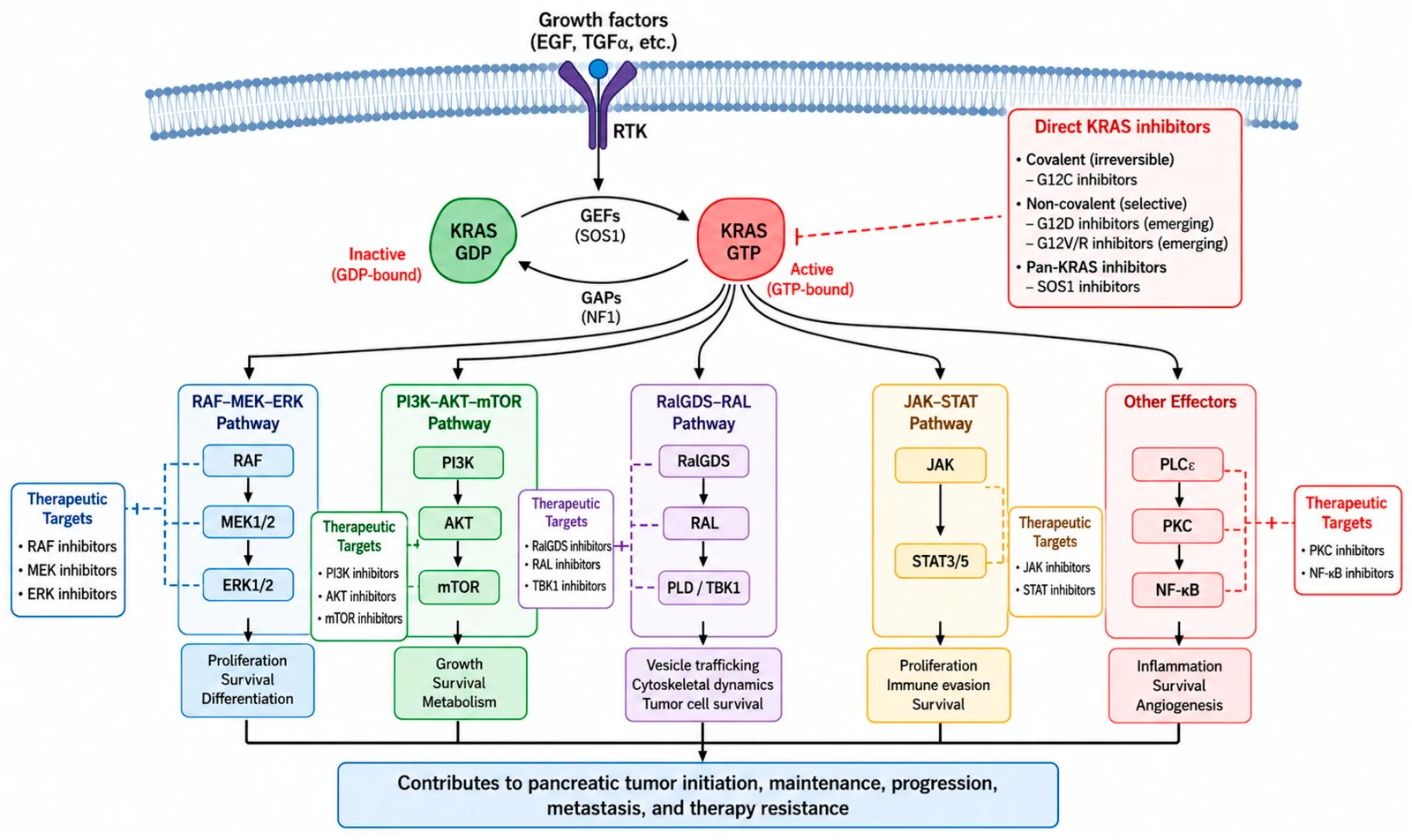
*KRAS* signaling pathways and therapeutic targets in PDAC. Activation of receptor tyrosine kinases promotes conversion of *KRAS* from the inactive GDP-bound state to the active GTP-bound state through guanine nucleotide exchange factors. Activated KRAS stimulates multiple downstream signaling pathways, including RAF-MEK-ERK, PI3K-AKT-mTOR, RalGDS-RAL, JAK-STAT, and PLCe-PKC-NF-kB. These pathways regulate tumor cell proliferation, survival, metabolism, inflammation, immune evasion, angiogenesis, metastasis, and treatment resistance. Potential therapeutic interventions include direct KRAS inhibitors and inhibitors targeting upstream regulators and downstream pathway components.

The major *KRAS* downstream signaling pathways and their corresponding therapeutic targets in PDAC are summarized in Figure 2.

### 2.3. Indirect KRAS-Directing Strategies

Before the development of direct inhibitors, therapeutic efforts were inclined towards interrupting the downstream signaling or modulating the upstream proteins. Some of these indirect approaches involved the following:

#### 2.3.1. Targeting Downstream Signaling

KRAS works by activating pathways such as RAF/MEK/ERK and PI3K/AKT/mTOR. The constitutive activation of these pathways can lead to tumor proliferation, forming the basis for therapeutic strategies targeting these pathways for the treatment of PDAC. However, several phase II studies of MEK inhibitors did not show efficacy as monotherapies for the treatment of PDAC including CI-1040, selumetinib, pimasertib, and trametinib [14]. The predominant explanation for these disappointing outcomes is the compensatory feedback activation and extensive crosstalk between the pathways that rapidly restore proliferative signaling [14].

The efficacy of treatment can be improved by identifying particular molecular subtypes and appropriate combination therapies to select the correct therapy for a particular patient. This was demonstrated by Mirzoeva et al. [15], who showed the effectiveness of combination therapy of epidermal growth factor receptor (EGFR) with MEK inhibitors for targeting the epithelial subtype of pancreatic carcinoma.

#### 2.3.2. Targeting Metabolic Dependencies and Synthetic Lethal Interactions

*KRAS*-mutant PDAC exhibits distinct metabolic reprogramming, including dependence on autophagy and macropinocytosis, along with significant alteration in nutrient processing pathways such as glutamine utilization [16,17]. Therefore, these metabolic dependencies create potentially actionable therapeutic targets in pancreatic cancer. Some examples of these include targeting multiple steps in the glutamine metabolism pathway, the most promising being the use of glutaminase inhibitors in order to directly target the breakdown of glutamine [18]. Another example is the use of autophagy inhibitors, which have shown promising results in mouse models and may have future effectiveness in the treatment of pancreatic cancer [19].

### 2.4. Direct KRAS Inhibition

**Figure 3:**
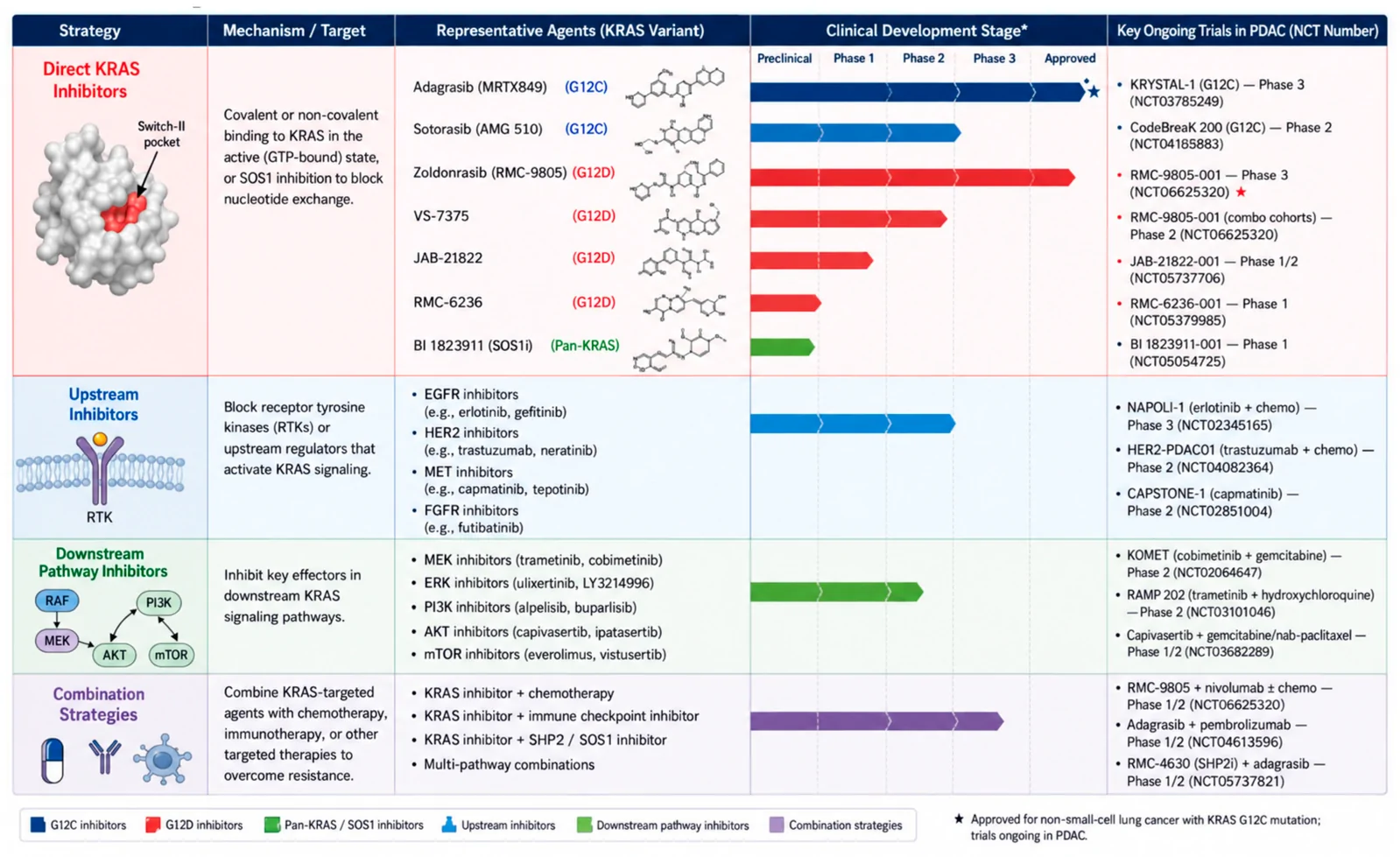
Therapeutic landscape of KRAS-directed strategies in pancreatic cancer. This figure summarizes major approaches under clinical and preclinical evaluation, including allele-specific KRAS inhibitors, multi-selective and pan-KRAS inhibitors, upstream receptor and regulatory protein inhibitors, downstream pathway inhibitors, and combination strategies. Representative agents, mechanisms of action, clinical development stages, and selected ongoing trials in PDAC are shown. Development stages reflect the most advanced reported stage across solid-tumor indications as of 29 July 2026 and may differ from the stage reached specifically in pancreatic cancer.

The breakthrough in KRAS directed therapy came with the identification of a previously unrecognized pocket adjacent to the nucleotide-binding site of KRAS-G12C that exists only in the inactive GDP-bound conformation and could act as site for binding of allosteric inhibitors [20]. Direct RAS inhibition strategies have evolved from allele-specific inhibitors like sotorasib and adagrasib, that covalently only targets KRAS G12C mutations, to pan KRAS inhibitors that reversibly bind multiple KRAS-mutant variants (G12D, G12V, and G13D) while sparing NRAS and HRAS, to Pan-RAS inhibitors that target all three Ras isoforms (KRAS, NRAS, HRAS) in their active GTP-bound state [21]. The current therapeutic landscape of KRAS-directed strategies in PDAC, including allele-specific inhibitors, multi-selective and pan-KRAS/RAS approaches, upstream and downstream pathway inhibitors, and combination strategies, is summarized in Figure 3.

#### 2.4.1. First-Generation KRAS-G12C Inhibitors: Sotorasib, Adagrasib

KRAS-G12C inhibitors were the first direct KRAS inhibitors to have successfully been used in the treatment of several tumors with *KRAS* mutations. Sotorasib was the first to enter clinical trials and receive FDA approval for *KRAS*-G12C-mutant non-small-cell lung cancer (NSCLC) [22]. The CodeBreaK 100 trial (NCT03600883) evaluated sotorasib in patients with pretreated *KRAS* G12C-mutant solid tumors, including 38 patients with pancreatic cancer [23,24]. The KRYSTAL-1 (NCT03785249) trial studied the efficacy of another KRAS-G12C inhibitor, adagrasib, which showed encouraging responses, but the data for PDAC were limited due to a small cohort [25]. However, the overall impact of KRAS-G12C inhibitors on PDAC treatment is limited due to low prevalence of *KRAS*-G12C mutation in PDAC (generally around 1–2% of cases reported in many cohorts), as well as the development of rapid resistance against these inhibitors [4].

#### 2.4.2. Second-Generation KRAS G12C Inhibitors

Glecirasib (JAB-21822) has demonstrated the most robust PDAC-specific data among next-generation KRAS G12C inhibitors. In a pooled analysis of two phase 1/2 trials, 32 PDAC patients were treated with glecirasib 800 mg once daily, achieving a confirmed ORR of 46.9% with a median PFS of 5.5 months and median OS of 10.8 months. However, it is important to note it was a Chinese-only study and enrolled patients with only one prior line of therapy [26].

Divarasib (GDC-6036) is another next-generation KRAS G12C inhibitor that binds to the Cysteine-12 residue and irreversibly locks *KRAS* in its inactive GDP-bound state. It was designed to have approximately 5–20 times the potency and up to 50 times greater selectivity than first-generation agents like sotorasib and adagrasib.

In a phase I multi-tumor study, divarasib demonstrated encouraging clinical activity in PDAC. Among seven patients with *KRAS* G12C-mutated PDAC, three achieved a partial response (43%), and four additional patients had stable disease. These early results suggest meaningful antitumor activity in this population [27].

In addition to glecirasib and divarasib, Table 1 includes olomorasib and MK-1084 to provide comparative context for other second-generation KRAS G12C inhibitors with reported PDAC-specific clinical data. Table 1 summarizes selected first- and second-generation KRAS G12C inhibitors, including their treatment setting, objective response rate, progression-free survival, overall survival, and study population.

Because the available data derive from non-randomized studies with differences in cohort size, prior treatment exposure, geographic enrollment, response assessment methods, and duration of follow-up, the efficacy estimates summarized in Table 1 are descriptive and should not be interpreted as head-to-head comparisons between agents.

#### 2.4.3. KRAS-G12D Inhibitors: MRTX1133 (Now Discontinued), RMC-9805, and GFH375

*KRAS* G12D is the most prevalent mutation in PDAC, accounting for more than 40% of cases, making it a critical therapeutic target [28]. But the lack of a binding pocket made this process difficult. Using structure-based design and optimization, Mirati successfully developed a selective non-covalent KRAS-G12D inhibitor called MRTX1133 [29,30]. Due to the initial promising results of the preclinical studies, early-phase clinical evaluation (phase I/II trials) was designed to further gauge the use of MRTX1133 for the treatment of tumors, including a large proportion of PDAC patients [31]. However, this phase I/II clinical trial of MRTX1133 (NCT05737706) was discontinued in early 2025 due to formulation and pharmacokinetic challenges following the acquisition of Mirati by Bristol Myers Squibb.

Another promising drug candidate for targeting KRAS-G12D is RMC-9805 (zoldonrasib), which is a mutation-selective covalent inhibitor that employs a unique tri-complex mechanism involving Cycophilin A (CYPA). The compound binds to CYPA and creates a neomorphic protein–protein interface with GTP-bound KRAS G12D, enabling inhibition of KRAS(ON) state [32].

Preclinical studies demonstrated marked antitumor activity and durable tumor regression in KRAS G12D-mutant PDAC xenografts and animal models [32]. RMC-9805 is currently in Phase 1/2 clinical trials (NCT06040541).

VS-7375, also known as GFH375 in China, is an oral KRAS G12D (ON/OFF) inhibitor licensed by Verastem Oncology from GenFleet Therapeutics. Preliminary findings from a Chinese dose-escalation study demonstrated favorable oral bioavailability, no observed dose-limiting toxicities across six dose levels, and early evidence of antitumor activity, including partial responses in patients with pancreatic and lung cancers [33].

Selected clinical efficacy data for emerging KRAS G12D-selective inhibitors and the multi-selective RAS inhibitor RMC-6236 in PDAC are summarized in Table 2, including dose, line of therapy, objective response rate, disease control rate, progression-free survival, and overall survival.

#### 2.4.4. RAS(ON) Multi-Selective Inhibition: Daraxonrasib (RMC-6236) and the RASolute 302 Trial

The randomized phase III RASolute 302 trial (NCT06625320) compared daraxonrasib with investigator-chosen standard-of-care chemotherapy in 500 patients with previously treated metastatic PDAC, of whom 91.8% had RAS G12 mutations [35]. In the RAS G12 population, daraxonrasib significantly improved median overall survival to 13.2 months compared with 6.6 months with chemotherapy (HR, 0.40; *p* < 0.001), and median progression-free survival to 7.3 months compared with 3.5 months (HR, 0.45; *p* < 0.001). Importantly, the magnitude of benefit was maintained in the overall study population, in which median overall survival was 13.2 versus 6.7 months (HR, 0.40; *p* < 0.001) and median progression-free survival was 7.2 versus 3.6 months (HR, 0.49; *p* < 0.001) [35]. The consistency of the overall survival benefit between the RAS G12 and overall populations, together with the similar progression-free survival effects, strengthens the evidence that multi-selective RAS(ON) inhibition can provide clinically meaningful benefit in previously treated metastatic PDAC. Notably, daraxonrasib approximately doubled median overall survival and progression-free survival compared with chemotherapy, representing a substantial improvement in a setting with limited effective treatment options. These findings distinguish daraxonrasib from allele-specific approaches, such as KRAS G12C inhibitors, by demonstrating the potential clinical value of targeting a broader range of RAS-driven tumors. However, because 91.8% of the overall study population had RAS G12 mutations, the overall population results should not be interpreted as definitive evidence of equivalent efficacy in the relatively small non-G12 or RAS-undetected subgroups. Taken together, the findings position daraxonrasib as a potentially broadly applicable RAS-directed treatment for previously treated metastatic PDAC and represent an important advance in the evolving therapeutic landscape of this disease.

#### 2.4.5. Critical Comparison of KRAS/RAS-Targeting Strategies in PDAC

The different approaches to KRAS/RAS inhibition should not be regarded as therapeutically equivalent, because their mutant coverage, dependence on the RAS nucleotide state, effects on wild-type RAS signaling, and susceptibility to adaptive resistance differ substantially [21,36]. Allele-specific KRAS(OFF) inhibitors provide the highest degree of molecular selectivity by targeting a defined mutant allele in its inactive GDP-bound state. This selectivity minimizes direct inhibition of wild-type RAS and may provide a favorable therapeutic window for combination therapy. However, their applicability is inherently limited by mutation frequency; this is particularly relevant in PDAC, in which *KRAS* G12C accounts for only a small minority of tumors. Furthermore, OFF-state inhibition depends on the mutant protein cycling into the GDP-bound state, and adaptive activation of receptor tyrosine kinase–SHP2–SOS1 signaling can increase RAS-GTP loading and reactivate wild-type RAS or downstream signaling, thereby reducing the depth and durability of pathway suppression [21,36].

Allele-specific KRAS(ON) inhibitors address part of this limitation by directly targeting the active GTP-bound mutant protein. This strategy may be particularly relevant for KRAS variants that predominantly occupy the active state and reduces dependence on nucleotide cycling into the GDP-bound conformation. Because these agents remain mutation-selective, they may retain an advantage in therapeutic window compared with broader RAS inhibition. Nevertheless, their activity remains restricted to the targeted allele, and neither ON-state nor OFF-state allele-specific inhibition eliminates the potential for secondary *KRAS* alterations, amplification, or bypass signaling through other RAS isoforms and parallel pathways [21,32,36].

Broader approaches trade some molecular selectivity for increased mutant coverage. Pan-KRAS inhibitors are designed to inhibit multiple mutant *KRAS* variants and may therefore cover the predominant G12D, G12V, and other *KRAS* alterations encountered in PDAC while avoiding direct inhibition of NRAS and HRAS. However, agents that preferentially bind the inactive *KRAS* state may remain dependent on nucleotide cycling, and inhibition of wild-type KRAS may also constrain the therapeutic window [21]. RAS(ON) multi-selective inhibitors such as daraxonrasib take this concept further by targeting the active GTP-bound state of multiple mutant and wild-type RAS isoforms [37,38]. This broader activity may suppress both the primary oncogenic RAS driver and compensatory signaling through wild-type RAS, providing a mechanistic rationale for greater pathway coverage than allele-specific inhibition. The trade-off is the potential for toxicity from inhibition of physiologic wild-type RAS signaling. Nevertheless, the manageable safety profile observed with daraxonrasib in early-phase studies and the significant overall survival and progression-free-survival benefits demonstrated in the phase III RASolute 302 trial indicate that clinically meaningful broad RAS inhibition can be achieved within a therapeutic window [34,35]. Importantly, this broader mechanism should not be interpreted as resistance-proof, because adaptive and acquired resistance can still emerge through alterations within or outside the RAS pathway [36].

In contrast, upstream and downstream pathway inhibition offers broad applicability without requiring a specific *KRAS* allele, but generally provides less tumor-specific pathway suppression. Inhibition of downstream MEK or PI3K signaling is vulnerable to pathway redundancy, relief of negative feedback, and compensatory signaling, which likely contributes to the limited efficacy observed with MEK inhibitor monotherapy in PDAC [14]. Upstream inhibition of SHP2 or SOS1 may reduce RAS activation and can complement direct KRAS inhibition, but these approaches also affect signaling from multiple receptor pathways and may be limited by tolerability and incomplete pathway suppression. Consequently, their most compelling role in PDAC may be as rational combination partners, rather than as stand-alone therapies [21,39].

Taken together, the optimal balance in PDAC is between mutant coverage and therapeutic selectivity. Allele-specific inhibitors offer precision, and potentially greater combinability, but treat narrower molecular subsets and remain vulnerable to pathway reactivation. Broad KRAS/RAS inhibitors increase population coverage, and may suppress compensatory RAS signaling, but potentially incur greater on-target toxicity through inhibition of wild-type proteins. At present, RAS(ON) multi-selective inhibition has the strongest evidence for near-term clinical impact in previously treated metastatic PDAC because daraxonrasib has demonstrated a survival benefit over chemotherapy in a randomized phase III trial [35]. Allele-specific G12D-directed approaches remain particularly important because of the high prevalence of this mutation and may ultimately offer a complementary strategy if durable efficacy can be achieved with a favorable therapeutic window. These considerations also provide a rationale for the combination strategies discussed below, which aim to deepen pathway suppression while limiting or delaying adaptive resistance.

#### 2.4.6. Patient Selection and Biomarker Consideration

The clinical implementation of KRAS-directed therapy requires molecular selection at the allele level. Comprehensive tumor genomic profiling can establish the specific *KRAS* alteration and identify additional genomic features that may influence treatment selection [40]. When adequate tumor tissue is available, tissue-based next-generation sequencing provides a comprehensive baseline molecular profile. Plasma circulating tumor DNA (ctDNA) provides a complementary approach when tissue is insufficient or repeat tissue sampling is impractical, particularly in metastatic disease. However, a negative plasma result should be interpreted cautiously because ctDNA detection in PDAC is strongly influenced by disease stage and tumor burden. In a large PDAC cohort, ctDNA detection was substantially higher in stage IV than in earlier-stage disease, while concordance for *KRAS* variants between plasma and tissue was high in untreated metastatic PDAC [41]. Thus, negative liquid biopsy testing should not necessarily exclude a potentially actionable *KRAS* alteration when adequate tissue testing remains feasible.

For allele-specific inhibitors, identification of the exact *KRAS* variant is essential because agents directed against G12C or G12D are not interchangeable across molecular subtypes. Broader RAS(ON) or multi-selective approaches may encompass a wider range of RAS alterations, but molecular characterization remains important for determining eligibility for individual trials and interpreting response. Co-occurring alterations in *TP53*, *SMAD4*, and *CDKN2A* provide additional information regarding PDAC biology and may influence disease behavior or resistance, although they are not currently validated biomarkers for selecting patients for KRAS inhibitors. In contrast, MTAP loss represents a specific biomarker for emerging MTA-cooperative PRMT5 strategies; for example, NCT06922591 requires MTAP loss for its PDAC treatment cohorts, with RAS mutation additionally required for the RAS inhibitor combination arms [42,43].

Serial ctDNA assessment may also have value beyond initial treatment selection. Changes in mutant allele burden during therapy are being investigated as pharmacodynamic markers of RAS pathway inhibition, and emerging genomic alterations in plasma may provide an opportunity to identify resistant clones before or at clinical progression [44,45]. Nevertheless, ctDNA-guided modification of KRAS-directed treatment remains investigational and has not yet been established as a standard clinical strategy. Similarly, transcriptional state, pathway activation, and other molecular features that may determine the degree of KRAS dependency or resistance are increasingly recognized in preclinical studies [46,47], but currently lack sufficient prospective validation for routine patient selection.

### 2.5. Combination Strategies

Despite promising clinical activity, KRAS inhibitors as monotherapies face significant limitations due to development of adaptive resistance mechanisms [46]. Hence, combination strategies are being actively investigated to enhance the durability of response of KRAS inhibitors in metastatic PDAC.

#### 2.5.1. KRAS Inhibitors with Chemotherapy

Combining KRAS inhibition with cytotoxic chemotherapy may enhance efficacy by targeting complementary vulnerabilities: KRAS blockade suppresses oncogenic signaling and proliferation, while chemotherapy induces DNA damage and apoptosis in tumor cells. In genetically engineered mouse models, the KRAS G12D inhibitor MRTX1133 combined with chemotherapy (gemcitabine and nab-paclitaxel) demonstrated superior tumor regression and metastasis prevention compared to either modality alone [47]. These findings provide compelling preclinical rationale for clinical translation. Researchers are actively planning and launching clinical trials to test different combinations, treatment sequences, and dosing strategies.

An early phase Ib/II study evaluated HRS-4642, a novel KRAS-G12D inhibitor, combined with gemcitabine and nab-paclitaxel in 31 patients with advanced KRAS-G12D-mutant pancreatic cancer. Among 30 treatment-naïve patients, the combination achieved a confirmed objective response rate of 60% and disease control rate of 93.3%. The regimen demonstrated a manageable safety profile with myelosuppression as the primary toxicity (grade ≥3 neutropenia in 61.3%), no treatment discontinuations due to adverse events, and no treatment-related deaths [48].

#### 2.5.2. KRAS Inhibitors with Immunotherapy

Immunotherapy represents a promising strategy with KRAS inhibitors, particularly given that Pancreatic cancer is typically resistant to immunotherapy alone. Immunotherapies, like checkpoint inhibitors and vaccines, can help modulate the tumor microenvironment and augment the effect of KRAS inhibitors. Preclinical studies showed enhanced T cell infiltration and activation, depletion of immunosuppressive myeloid cells, and increased antigen cross-presentation by dendritic cells within the tumor core, leading to complete tumor resolution and prolonged survival times [49]. Studies in KRAS-mutant tumor models have also shown that KRAS inhibition can downregulate immunosuppressive cytokines, reduce the recruitment and function of myeloid-derived suppressor cells and tumor-associated macrophages, and enhance antigen presentation [50]. Although these findings are not specific to PDAC, they provide mechanistic support for investigating KRAS inhibition in combination with immunotherapy in the PDAC tumor microenvironment.

Another immunologic approach involves the development of mRNA vaccines from surgically resected PDAC tumors. These vaccines have shown the ability to induce potent and specific T cell responses against *KRAS* neoantigens, with some evidence of clinical activity in patients with PDAC [51]. The combination of KRAS inhibitor with KRAS-specific vaccine may generate durable memory responses, offering potential for long-term disease control.

#### 2.5.3. Combination of Multi-RAS Inhibitors with Allele-Specific KRAS Inhibitors

The combination of Multi-Ras (Pan-RAS) inhibitors with allele-specific KRAS inhibitors represents an emerging strategy to overcome resistance and achieve deeper pathway suppression. Preclinical data from *KRAS* G12C-mutant NSCLC models demonstrate that broad RAS-GTP inhibition can overcome resistance to allele-specific KRAS inhibitors, such as sotorasib and adagrasib, arising from pathway reactivation, including *KRAS* amplification or secondary RAS alterations [52]. Although these findings were generated outside PDAC, they provide a mechanistic rationale for evaluating analogous combination strategies in pancreatic cancer, where adaptive RAS pathway reactivation is also an important mechanism of resistance.

Clinically, RMC-6236 (aRAS(ON) multi-selective inhibitor) has demonstrated a disease control rate of 85–87% as monotherapy in NSCLC and PDAC in early phase trials. NCT06040541 is an early phase trial evaluate the safety and tolerability of RMC-9805 as monotherapy and in combination with RMC-6236 in adults with *KRAS* G12D-mutant solid tumors.

#### 2.5.4. KRAS Inhibitors and MTA—Cooperative PMRT5 Inhibitor Combination

MTAP is located on Chromosome 9p21, directly next to tumor suppressor gene CDKN2A, and is often lost as part of common 9p21 deletion seen across many cancers. Approximately 22% of PDAC cases harbor co-deletion of methylthioadenosine phosphorylase (MTAP) gene alongside CDKN2A mutation, a genomic event that creates a unique therapeutic vulnerability [42]. This deletion leads to the accumulation of methylthioadenosine (MTA), which acts as the partial inhibitor of protein arginine methyltransferase 5 (PRMT5). This renders the MTAP-deficient tumors selectively sensitive to MTA-cooperative PRMT5 inhibitors, which may synergize with *KRAS* blockade. Hence, this combination therapy is being tested as a potential treatment modality.

The preclinical rationale for combining KRAS/RAS-directed therapy with MTA-cooperative PRMT5 inhibition has now progressed into early clinical evaluation. Recent CRISPER-based screening studies have identified KRAS inhibition as a highly synergistic combination partner with MTA-cooperative PRMT5 inhibitors in MTAP-deleted PDAC, with combination therapy leading to greater and more durable response in MTAP-deleted, KRAS-mutant PDAC preclinical models [53].

Several MTA-cooperative PRMT5 inhibitors are currently in early phase clinical trials, including AZD3470 (NCT06130553), TNG 462 and BMS-986504. The convergence of PRMT5 and *KRAS* on distinct yet complementary pathways governing PDAC growth, combined with non-cross-reactive resistance mechanism, provides strong rationale for further clinical evaluation of this combination strategy.

NCT06922591 (TNG462-C102) is a Phase 1/2 trial evaluating TNG462 (a PRMT5/MAT2A inhibitor) combined with RMC-6236 in patients with MTAP-deleted cancers, including pancreatic ductal adenocarcinoma. Additional arms evaluate TNG462 with standard chemotherapy (mFOLFIRINOX or gemcitabine/nab-paclitaxel). The trial is currently recruiting patients with ECOG 0–1 who have received prior standard therapy, but no prior PRMT5, MAT2A, or RAS-targeted agents.

#### 2.5.5. Targeting the Tumor Microenvironment

Co-targeting *KRAS* and stromal elements (FAK inhibitors, hedgehog pathway inhibitors) aims to disrupt the physical and biochemical barriers of the tumor microenvironment, improving drug delivery and reducing signaling from cancer-associated fibroblasts [54]. Preclinical studies demonstrated that FAK inhibition, combined with RAF–MEK reprograms stromal fibroblasts, reduces tumor growth factor signaling and increases the cytotoxic effect of chemotherapy [55].

Early clinical data from the ACCENT trial (NCT05355298) evaluating Narafotinib (A FAK inhibitor) combined with gemcitabine/nab-paclitaxel showed ORR of 31% among 55 patients with acceptable tolerability [56].

### 2.6. Clinical Trials Targeting KRAS/RAS-Directed and Mechanism-Based Strategies Relevant to PDAC

Ongoing clinical development in PDAC encompasses several mechanistically distinct approaches, including allele-specific KRAS inhibition, multi-selective or pan-RAS/KRAS inhibition, KRAS-directed immunotherapy and vaccines, combination strategies designed to achieve broader pathway suppression, and synthetic lethal approaches [57]. Selected trials are organized by therapeutic mechanism in Table 3, with the PDAC-specific nature of each study or cohort indicated separately.

### 2.7. Resistance Mechanisms to KRAS-Directed Therapy

A major challenge with KRAS inhibitors, particularly as monotherapies, is the inevitable development of resistance. Resistance can arise through both mutational and non-mutational mechanisms, with the resistance-causing mutations occurring not only in the *KRAS* gene, but also in non-KRAS genes [36].

It has been seen in patients with KRAS-G12C-mutant PDAC treated with adagrasib or sotorasib that acquired resistance can be linked to mutations in KRAS and PIK3CA, as well as amplifications of KRAS-G12C, MYC, MET, EGFR, and CDK6 [58]. In preclinical models, including PDAC cell lines and organoids exposed to the KRAS-G12D inhibitor MRTX1133, resistance was associated with activation of PI3K–AKT–mTOR signaling and induction of epithelial-to-mesenchymal transition [58].

**Figure 4:**
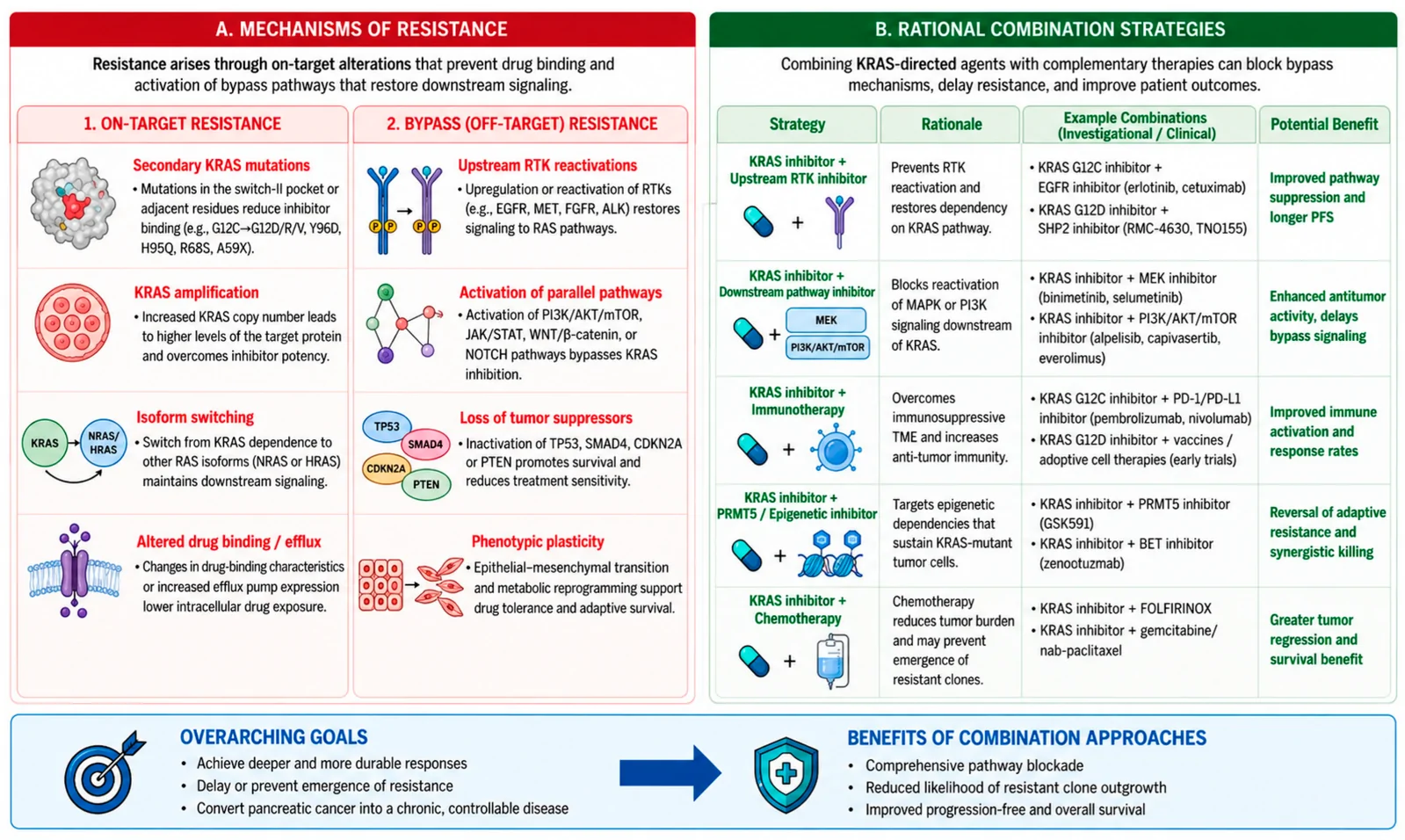
Mechanisms of resistance to KRAS-directed therapies and rational combination strategies in PDAC. Resistance to KRAS inhibition may develop through on-target mechanisms, including secondary *KRAS* mutations, *KRAS* amplification, isoform switching, and altered drug binding, as well as bypass mechanisms involving receptor tyrosine kinase reactivation, parallel signaling pathways, loss of tumor suppressor function, and phenotypic plasticity. Rational combination approaches pair KRAS-directed agents with upstream or downstream pathway inhibitors, immunotherapy, epigenetic, or PRMT5-targeted agents, and chemotherapy to achieve more complete pathway suppression, delay the emergence of resistant clones, and improve the depth and durability of treatment response.

Ash and colleagues have presented two broad classes of resistance mechanisms for KRAS-directed therapy: direct (on-target) and indirect (off-target or bypass) [36]. Direct mutations include mutations in the KRAS gene itself. These mutations can act to alter the binding site, leading to reduced binding of the drug, or they can cause gene amplification, causing increased number of the mutant *KRAS* genes, leading to their overexpression. The indirect or bypass mechanisms can include mutations in non-KRAS genes that allow growth, even when *KRAS* is inhibited, by restoring downstream signaling or activating parallel pathways. They can also include some non-genetic mechanisms, such as changes in transcriptional programs or epigenetic modifications, which allow tumor cells to adapt their dependency away from *KRAS* signaling [36]. The major on-target and bypass mechanisms of resistance to KRAS-directed therapies, together with corresponding rational combination strategies, are summarized in Figure 4.

### 2.8. Overcoming the Resistance Mechanisms

Recent studies have shown that combination therapies can help counter the problem of KRAS therapy resistance. The optimal combination strategy depends on the underlying mechanism of resistance (Figure 4). On-target alterations such as secondary *KRAS* mutations, *KRAS* amplification, or reactivation through alternative RAS isoforms provide a rationale for broader RAS(ON) inhibition or combined allele-specific and multi-selective RAS blockade. In contrast, adaptive receptor tyrosine kinase–SHP2 signaling can be addressed by combining KRAS inhibition with upstream SHP2 inhibition, whereas activation of PI3K–AKT–mTOR signaling supports co-targeting of PI3K/AKT or mTOR pathways [39,59,60]. Non-genetic resistance mechanisms, including phenotypic plasticity and remodeling of the tumor microenvironment, provide a rationale for combinations with chemotherapy, immunotherapy, or microenvironment-directed approaches, as discussed in Section 2.5. Thus, resistance mechanisms should not be viewed as a single phenomenon, but rather as biologically distinct escape routes that require mechanism-matched combination strategies. For example, Brown et al. showed that, in preclinical PDAC models, the inhibition of *KRAS* or *MEK* leads to adaptive activation of the mTORC2/AKT pathway through integrin-linked kinase-mediated phosphorylation of Rictor, thereby sustaining tumor survival. Dual blockade of *KRAS* (or *MEK*), together with mTORC1/2 inhibition, helped restore sensitivity, producing synergistic cytotoxicity and durable tumor control [59].

Other studies support co-targeting of PI3K/AKT signaling along with KRAS or MAPK inhibitors. Co-targeting caused a synergistic anti-tumor response, helping to overcome drug resistance and improve efficacy [60]. Another study worked on the inhibition of SHP2, a tyrosine phosphatase upstream of RAS. Yaeger et al. demonstrated that combining KRAS inhibitors with SHP2 inhibitors overcomes adaptive resistance in preclinical models by blocking the reactivation of upstream receptor signaling that would otherwise circumvent KRAS inhibition [39]. These examples show promising results regarding tackling the problem of drug resistance, suggesting that such combination strategies may represent the path forward for future therapies.

## 3. Conclusions

The development of KRAS-directed therapies represents a paradigm shift in the treatment of pancreatic ductal adenocarcinoma, a disease long defined by its therapeutic intractability. After decades of failed attempts to target what was considered an “undruggable” oncoprotein, the clinical validation of direct KRAS inhibition has fundamentally altered the therapeutic landscape. The ongoing clinical trials are essential for establishing the efficacy of these novel treatments, which are poised to become cornerstone of precision oncology for PDAC, offering renewed hope for patients with this devastating malignancy. Notably, the phase III RASolute 302 trial demonstrated significantly improved overall and progression-free survival with daraxonrasib compared with standard-of-care chemotherapy in previously treated metastatic PDAC, providing randomized evidence for the clinical efficacy of broad RAS(ON) inhibition. Meanwhile, G12D-selective approaches remain under active clinical investigation and may further expand the role of KRAS-directed therapy in PDAC. Among the approaches reviewed, RAS(ON) multi-selective inhibition currently has the strongest clinical evidence for near-term impact in previously treated metastatic PDAC, given its broad molecular coverage and demonstrated phase III survival benefit. In contrast, allele-specific G12D inhibitors may offer greater molecular selectivity and potentially greater flexibility for combination therapy, but still require validation of durable clinical benefit, whereas upstream and downstream pathway inhibitors appear more likely to serve as combination partners than as effective stand-alone strategies.

## Figures and Tables

**Table 1:** Selected KRAS G12C inhibitors with PDAC-specific clinical efficacy data available through 29 July 2026.

Agent	Evidence Source	Dose	PDAC Cohort	Prior Therapy	ORR	Median PFS	Median OS
Sotorasib	Peer-reviewed; Strickler et al., NEJM 2023 [23]	960 mg QD	N = 38	Median 2 prior lines (range 1–8)	8/38 (21%)	4.0 mo	6.9 mo
Adagrasib	Peer-reviewed; Bekaii-Saab et al., JCO 2023 [25]	600 mg BID	N = 21	Median 2 prior lines	7/21 (33.3%)	5.4 mo	8.0 mo
Divarasib	Peer-reviewed; Sacher et al., NEJM 2023 [27]	400 mg QD	N = 7	PDAC-specific prior-line distribution NR	3/7 (42.9%)	NR	NR
Glecirasib	Peer-reviewed; Li et al., Cancer Commun. 2025 [26]	800 mg QD	N = 32 efficacy-evaluable	Previously treated; PDAC-specific median prior lines NR	15/32 (46.9%)	5.5 mo	10.8 mo

Abbreviations: BID, twice daily; ORR, objective response rate; OS, overall survival; PDAC, pancreatic ductal adenocarcinoma; PFS, progression-free survival; QD, once daily; NR, not reported. Note: Cross-trial comparisons are descriptive only because cohort size, prior treatment exposure, response assessment method, geographic enrollment, follow-up duration, and data maturity differ among studies. Sotorasib and adagrasib response estimates were centrally assessed; divarasib PDAC responses were reported within a multi-tumor phase I cohort; glecirasib values are from the pooled PDAC efficacy-evaluable population.

**Table 2:** Selected emerging KRAS G12D-directed and RAS(ON) multi-selective agents with PDAC clinical efficacy data available through 29 July 2026.

Agent	Target	Evidence Source	Dose	PDAC Cohort	Prior Therapy/Setting	ORR	DCR	Median PFS	Median OS
Daraxonrasib (RMC-6236)	RAS(ON) multi-selective	Peer-reviewed; Wolpin et al., NEJM 2026 [34]	300 mg QD	N = 26 (defined efficacy subgroup)	Second-line, RAS G12-mutant PDAC	35%	NR	8.5 mo	13.1 mo
Zoldonrasib (RMC-9805)	KRAS G12D(ON)-selective	Conference abstract; ASCO GI 2025	1200 mg total daily (1200 mg QD or 600 mg BID)	N = 40	Previously treated; line NR	30%	80%	NR	NR
INCB161734	KRAS G12D inhibitor	Conference abstract; ASCO GI 2026	1200 mg total daily (QD or BID)	N = 29 efficacy-evaluable	Previously treated/advanced; line NR	10/29 (34%)	25/29 (86%)	NR	NR
HRS-4642	KRAS G12D inhibitor	Conference presentation; ESMO 2025 (915O)	200 mg QW–1200 mg Q2W	N = 24	Median 2 prior lines	20.8%	79.2%	NR *	NR
GFH375 (VS-7375)	KRAS G12D (ON/OFF) inhibitor	Conference presentation; ESMO 2025 (LBA84); sponsor disclosure	600 mg QD	N = 66 treated; N = 59 efficacy-evaluable	≥2L population; 68.2% had ≥2 prior lines	24/59 (40.7%)	57/59 (96.7%)	5.52 mo	NR †

* The ESMO 2025 report described a median PFS of 4.4 months in the 1200 mg Q2W HRS-4642 subgroup; this dose-specific estimate is not mixed with the all-cohort ORR/DCR values in the main table. † Median OS was not reported for GFH375/VS-7375; the reported 4-month OS rate was 92.2%. Abbreviations: BID, twice daily; DCR, disease control rate; ORR, objective response rate; OS, overall survival; PDAC, pancreatic ductal adenocarcinoma; PFS, progression-free survival; Q2W, once every 2 weeks; QD, once daily; QW, once weekly; NR, not reported. Note: Conference-only data are explicitly identified. Cross-trial comparisons are descriptive only because dose level, treatment line, eligibility criteria, efficacy-evaluable denominators, response confirmation, follow-up duration, and data maturity differ among studies.

**Table 3:** Selected ongoing clinical trials of KRAS/RAS-directed and mechanism-based therapeutic strategies relevant to PDAC, categorized by mechanism and with PDAC-specific study or cohort status indicated. Trial information and recruitment status were verified against ClinicalTrials.gov as of 29 July 2026.

Trial (NCT)	Drug/Intervention	Phase	Population	Status	Study Start Date
NCT06411691	KRAS-targeted long peptide vaccine + balstilimab + botensilimab	Phase 1 (1b study)	MMR-p metastatic CRC or PDAC after first-line chemotherapy	Recruiting	24 June 2025
NCT05669482	Avutometinib + defactinib + gemcitabine/nab-paclitaxel (RAMP 205)	Phase 1b/2a	Previously untreated metastatic PDAC	Active, not recruiting	22 March 2023
NCT07094113	AMG 410 ± pembrolizumab ± panitumumab	Phase 1/1b	KRAS-altered advanced/metastatic solid tumors, including PDAC	Recruiting	31 July 2025
NCT05379985	Daraxonrasib (RMC-6236)	Phase 1/2	RAS-mutant advanced solid tumors, including PDAC, NSCLC, and CRC	Recruiting	31 May 2022
NCT06445062	RAS(ON) inhibitors (RMC-6236/RMC-9805) ± 5-fluorouracil-based regimens, cetuximab, gemcitabine, or nab-paclitaxel	Phase 1/2	Gastrointestinal solid tumors, including CRC and PDAC	Recruiting	24 May 2024
NCT07020221	VS-7375 ± cetuximab or standard chemotherapy/immunotherapy combinations	Phase 1/2a	KRAS G12D-mutated solid tumors, including PDAC, NSCLC, and CRC	Recruiting	24 June 2025
NCT06625320	Daraxonrasib (RMC-6236) vs. investigator-choice chemotherapy (RASolute 302)	Phase 3	Previously treated metastatic PDAC	Active, not recruiting	16 October 2024
NCT06015724	Daratumumab + KRAS vaccine + nivolumab	Phase 2	Advanced PDAC or refractory NSCLC	Recruiting	31 January 2024
NCT03785249	Adagrasib (MRTX849) ± pembrolizumab/cetuximab/afatinib (KRYSTAL-1)	Phase 1/2	Advanced solid tumors with KRAS G12C mutation	Active, not recruiting	15 January 2019
NCT06447662	PF-07934040 ± standard chemotherapy/immunotherapy combinations	Phase 1	Advanced solid tumors with KRAS mutation, including PDAC, CRC, and NSCLC	Active, not recruiting	27 June 2024
NCT06895031	JYP0015 (pan-RAS inhibitor)	Phase 1/2	RAS-mutant advanced solid tumors, including PDAC, NSCLC, and CRC	Recruiting	31 March 2025
NCT06782932	AGEN2373 + balstilimab + GVAX or mKRASvax	Phase 1/2	Surgically resectable pancreatic adenocarcinoma	Recruiting	27 May 2025
NCT06040541	Zoldonrasib (RMC-9805) ± daraxonrasib (RMC-6236)	Phase 1/1b	KRAS G12D-mutant solid tumors, including PDAC, NSCLC, and CRC	Recruiting	7 September 2023
NCT06917079	BBO-11818 ± pembrolizumab/chemotherapy/cetuximab	Phase 1a/1b	Locally advanced/metastatic KRAS-mutant solid tumors, including PDAC, NSCLC, and CRC	Recruiting	31 March 2025
NCT06818812	INCB186748 ± cetuximab/gemcitabine-nab-paclitaxel/mFOLFIRINOX	Phase 1	Advanced/metastatic solid tumors with KRAS G12D mutation	Active, not recruiting	27 March 2025
NCT06835569	ALTA3263 ± combination therapy	Phase 1/1b	Advanced solid tumors with KRAS mutations, including PDAC, NSCLC, and CRC	Recruiting	5 March 2025
NCT06179160	INCB161734 ± cetuximab/retifanlimab/gemcitabine-nab-paclitaxel/mFOLFIRINOX	Phase 1	Advanced/metastatic solid tumors with KRAS G12D mutation	Recruiting	4 January 2024
NCT06128551	Elironrasib (RMC-6291) ± daraxonrasib (RMC-6236)	Phase 1b/2	Advanced KRAS G12C-mutant solid tumors, including PDAC, NSCLC, and CRC	Recruiting	14 November 2023
NCT07023731	ARV-806	Phase 1/2	Advanced solid tumors with KRAS G12D mutation	Active, not recruiting	29 May 2025
NCT05462717	Elironrasib (RMC-6291)	Phase 1/1b	Advanced KRAS G12C-mutant solid tumors, including PDAC, NSCLC, and CRC	Active, not recruiting	19 September 2022
NCT06922591	TNG462 ± RMC-9805 or RMC-6236; additional chemotherapy combinations	Phase 1/2	MTAP-deleted cancers with RAS mutations, including PDAC and NSCLC	Recruiting	31 May 2025

ClinicalTrials.gov verification cutoff: 29 July 2026. Trial status is dynamic and may change after the stated cutoff. Removed from the prior version: NCT06704724 because it had been terminated by the cutoff; NCT06137144 because it concerns hematologic malignancies, rather than PDAC/solid-tumor KRAS-directed therapy. Abbreviations: CRC, colorectal cancer; MMR-p, mismatch repair-proficient; MTAP, methylthioadenosine phosphorylase; NSCLC, non-small-cell lung cancer; PDAC, pancreatic ductal adenocarcinoma.

## Data Availability

No new data were created or analyzed in this study. Data sharing is not applicable to this article.
